# The impact of age on burnout and secondary traumatic stress: Examining the moderating roles of detachment and work hours among mental health professionals serving war refugees in Pakistan

**DOI:** 10.1177/00207640251355840

**Published:** 2025-08-06

**Authors:** Momina Khalid Butt, Neringa Grigutytė, Adelė Petraviciutė, Jonas Eimontas

**Affiliations:** Department of Clinical Psychology, Institute of Psychology, Faculty of Philosophy, Vilnius University, Lithuania

**Keywords:** Secondary traumatic stress, burnout, detachment, mental health professional, refugees

## Abstract

**Background:**

With ongoing conflicts worldwide, the refugee crisis has escalated into a global humanitarian crisis, straining mental health professionals supporting war refugees. Growing demands and the emotional toll of witnessing trauma of the survivors of war highlight the need for research to better equip these professionals.

**Aims:**

This study examines influence of age on burnout and secondary traumatic stress (STS) among 103 mental health professionals working with war refugees in Pakistan, focusing on the moderating roles of detachment and work hours.

**Method:**

In this cross-sectional study, 103 mental health professionals completed the Professional Quality of Life Scale, Experiences Questionnaire, and Secondary Traumatic Stress Scale. Regression, moderation, and mediation analyses were conducted using SPSS PROCESS macro.

**Results:**

Increased work hours intensified the burnout–STS relationship, while detachment served as a protective factor by moderating this relationship rather than directly predicting STS. Additionally, burnout mediated the relationship between age and STS, with older professionals reporting lower burnout and, in turn, lower STS symptoms.

**Conclusions:**

These findings highlight the importance of addressing burnout in efforts to reduce STS among refugee-serving professionals and suggest that fostering detachment and managing work hours may provide protective benefits.

## Introduction

In today’s world, the refugee crisis has escalated into a global humanitarian crisis. By May, 2024, the UNHCR reported that 120 million people had been forcibly displaced from their homelands due to war and armed conflicts (USA for UNHCR, 2024). Refugees are defined as individuals who, ‘owing to a well-founded fear of being persecuted for reasons of race, religion, nationality, membership of a particular social group, or political opinion, are outside the country of their nationality and are unable or, owing to such fear, unwilling to avail themselves of the protection of that country’ (Herlihy et al., 2024). The traumatic experiences endured in conflict zones leave deep psychological scars, leading to a range of mental health issues. Approximately 45% of these individuals grapple with anxiety, and an even greater proportion 33.6% experience depression. The complexities of post-traumatic stress disorder (PTSD) are particularly alarming, affecting 37.3% of war refugees, with symptoms that often persist long after resettlement in peaceful environments (Melese et al., 2024). Another systematic review of studies involving nearly 7,000 war refugees residing in Western countries revealed that the prevalence rate of PTSD was as high as 86%, while depression affected up to 80% of this population (Aysazci-Cakar et al., 2022).

Amid these staggering statistics, the responsibility of addressing the mental health needs of this population falls heavily on the shoulders of mental health professionals who work closely with refugees.

Research focusing specifically on mental health professionals working with refugees has shown that these providers face unique stressors compared to those serving general trauma-affected populations, due to the chronicity of refugee trauma, cultural barriers, and resource limitations (Geiling et al., 2021; Wirth et al., 2019). Furthermore, the cumulative exposure to refugees’ stories of prolonged displacement, loss, and violence increases professionals’ vulnerability to secondary traumatic stress (STS) and burnout (Kanagaratnam et al., 2017).

The emotional and psychological toll of caring for traumatized individuals can lead to STS among these professionals, potentially impacting both their well-being and their ability to provide effective care. STS, which mirrors the symptoms of PTSD, occurs when caregivers absorb the emotional trauma of those they treat. According to previous findings, 15% to 39% of mental health professionals who work with traumatized populations develop Secondary Traumatic Stress (Bride, 2007; Dominguez-Gomez & Rutledge, 2009; Hatcher et al., 2011). This not only compromises their well-being but also impacts care quality (Acquadro Maran et al., 2023). As the psychological burdens of refugee care intensify, understanding the interplay of factors that contribute to STS has become critical.

One of the previously identified variable effecting STS amongst many populations is job burnout (Galek et al., 2011). Burnout, a state of emotional exhaustion, cynicism, and reduced professional efficacy is a pervasive issue in caregiving professions, particularly in those working with traumatized individuals (Maslach et al., 2001). However, the factors that influence the strength of this relationship, especially among professionals working with war refugees, remain underexplored. This raises critical questions about what moderates the relationship between burnout and STS, and which factors either mitigate or exacerbate the risks professionals face.

Age and detachment are two key variables examined in this study, both grounded in theoretical perspectives on emotional regulation and occupational resilience.

Previous research has consistently identified age as a crucial variable in understanding susceptibility to burnout and stress across various professional settings (Ramos et al., 2016). Theories of emotional regulation suggest that with age and professional maturity, individuals develop greater cognitive and emotional control, which may contribute to enhanced resilience against occupational stressors (Gross, 2015). Older and more experienced professionals may possess refined coping mechanisms, such as better emotional regulation, professional detachment, and meaning-making strategies (Gross, 2015)(Folkman & Moskowitz, 2004), which enhance their emotional resilience. In turn, this resilience can help buffer against burnout, thereby indirectly reducing susceptibility to STS (Gómez-Urquiza et al., 2017; Schwartz et al., 2007). However, in refugee-serving contexts, where providers often face unrelenting emotional demands, the protective effects of experience and resilience require further empirical examination.

In contrast, younger professionals, particularly those new to trauma-focused work, might lack the experience and coping strategies needed to handle the emotional toll, placing them at higher risk for both burnout and STS (Brewer & Shapard, 2004; Cheng et al., 2013). Despite these theoretical insights, little empirical research has directly examined how age functions in relation to burnout and STS within trauma-exposed populations, particularly among mental health workers in refugee contexts.

Another critical factor in this equation is detachment, which refers to the ability to psychologically distance oneself from work during non-working hours (Sonnentag & Kruel, 2006). In high-stress professions, detachment serves as a vital coping mechanism, enabling individuals to mentally recover from the emotional and cognitive demands of their work (Shimazu et al., 2016). Detachment is also conceptually linked to theories of boundary management and psychological recovery, which emphasize the role of work–life separation in maintaining occupational health (Sonnentag & Fritz, 2015). Despite the well-documented benefits of detachment in various high-stress professions, its role within the context of mental health care for war refugees remains relatively underexplored.

This study seeks to investigate the effects of detachment on STS, examining both its direct and indirect influences. It is hypothesized that in this population, detachment will have a direct negative relationship with STS, consistent with findings from previous studies (Beermann & Wöhrmann, 2018; Sonnentag & Niessen, 2020). Additionally, detachment is expected to play an indirect role by moderating the relationship between burnout and STS. It is hypothesized that detachment will weaken the positive relationship between burnout and STS, acting as a protective buffer. This potential buffering effect of detachment highlights its importance as a strategy for mitigating the emotional consequences of working in trauma-laden environments.

In contrast, the role of work hours is expected to amplify the relationship between burnout and STS (Hu et al., 2016). The number of hours professionals spend working directly with traumatized individuals is likely to increase their emotional exposure, intensifying the risks of burnout and eventually STS (Jovanović et al., 2016; Lin et al., 2021). This study examines work hours as a second moderator, hypothesizing that the more time professionals spend in direct contact with refugees, the stronger the link between burnout and STS becomes. These findings could have significant implications for policy recommendations on workload management in trauma-focused mental health professions.

It is important to consider that this study was conducted in Pakistan, a country that has hosted one of the largest and most prolonged refugee crises in the world, primarily due to decades of conflict in Afghanistan. Since the Soviet invasion of Afghanistan in 1979, the subsequent civil war, and more recent U.S.-led interventions, Pakistan has provided refuge to an estimated 3 million Afghan refugees, making it the second-largest refugee-hosting nation globally. The continuous influx of Afghan refugees over the past 40 years has placed a significant strain not only on the refugees themselves but also on the mental health professionals who provide care to them. Understanding how age, detachment, and work hours interact with burnout and STS is therefore particularly urgent in this high-risk context. This research examines both direct and moderating effects of age, detachment, and work hours on the burnout–STS pathway among Pakistani mental health professionals working with war refugees. By drawing on theories of emotional regulation and occupational resilience, this study seeks to identify both protective and risk factors to guide interventions. This understanding is crucial not only for the professionals’ well-being but also for ensuring continued quality care for displaced populations affected by global conflict.

Finally, this study addresses a significant research gap. Although STS has been widely studied in Western contexts, limited research has explored its impact on professionals in South Asia, particularly those supporting displaced populations. This investigation thus contributes novel insights into the psychological demands placed on mental health workers operating in conflict-affected, resource-constrained settings.

## Methodology

### Participants

The participants in this study were mental health professionals, including psychologists, psychiatrists, and social workers, who were actively involved in providing support services to war refugees in Pakistan. The inclusion criteria required that participants were directly working with refugees and willing to participate voluntarily. A purposive sampling strategy was employed, aiming to recruit individuals with specific experience in providing mental health care to refugees.

A total of 111 participants initially took part in the study. However, after applying exclusion criteria, eight participants were removed: three participants reported they were not directly working with refugees, and five participants were excluded due to incomplete responses (more than 50% of the survey left unanswered). The final sample comprised 103 participants (55 females and 56 males). The demographic characteristics of the sample, including age, gender, marital status, education, and work experience, are summarized in Table 1.

**Table 1.** Demographic characteristics of the sample (*N* = 103 for Pakistan).

Characteristics	*M*	*SD*
Age	34.77	9.22
Weekly hours of working with refugees	26.28	13.04
	*n*	*%*
Sex		
Female	55	49.5
Male	56	50.5
Level of education		
Secondary	2	1.8
Vocational	5	4.5
Higher	96	93.2
Occupation		
Psychotherapists	24	23.3
Psychologists	35	33.9
Social workers and their assistants	25	24.2
Other (psychiatry nurses, psychiatry interns, and psychology student)	19	18.4
Experience of working with refugees (years)		
<1	6	6.18
1–2	11	11.3
2–3	15	15.4
3–4	21	19.1
>5	50	51.5

Note. *M* = mean; *SD* = standard deviation.

### Procedure

Data was collected using an online survey administered through Google Forms. Invitations were distributed via email and professional networks to individuals working in mental health facilities, private clinics, NGOs, and refugee centers across Pakistan. Additionally, professional contacts were encouraged to share the survey with eligible colleagues, creating a snowball sampling effect. Before presenting the detailed questionnaire, informed consent containing all necessary information regarding the study was provided to the participants. The detailed questionnaire included questions to gather demographic information such as participants’ age, gender, and working hours with refugees, along with the scales to assess burnout, detachment, and STS. Participation was voluntary, and confidentiality and anonymity were assured to all respondents. To ensure confidentiality, identifiable data (such as names and email addresses) were stored separately in a secure location, accessible only to the first author and research supervisor. The dataset used for statistical analysis did not contain any identifying information; all personal identifiers were removed prior to analysis to maintain participant anonymity. Before beginning of the research, ethical clearance was obtained from the concerned Ethical Committee of the research institute. Also, permissions from the authors were obtained before using all instruments.

### Instruments

#### Demographic variables

Participants were asked to fill out their personal information namely, their gender, age, profession, and years of experience in working with war refugees and their weekly hours of working with war refugees.

#### Professional Quality of life scale

To measure burnout among participants, ProQOL was used (Hudnall Stamm, 2010). The ProQOL consists of 30 items, answered on a scale of 1 (never), 2 (rarely), 3 (sometimes), 4 (often), or 5 (very often). Participants rate how frequently each item was experienced in the past 30 days. The ProQOL is comprised of three subscales: compassion satisfaction (10 items), burnout (10 items), and compassion fatigue (10 items). Only the Burnout subscale was used to measure burnout in this study. A score below 18 indicates low burnout, 19 to 26 indicates average burnout, and scores above 27 suggest high burnout risk. In the current sample, the Cronbach’s alpha for the Burnout subscale was .72, indicating acceptable internal consistency. Importantly, STS was not measured using the ProQOL Compassion Fatigue subscale; instead, STS was measured independently using the full Secondary Traumatic Stress Scale (STSS; see Secondary Traumatic Stress Scale Section), following best practices for conceptual clarity and clinical relevance.

This scale has been widely used in humanitarian and trauma-focused professions and is suitable for the Pakistani refugee care context due to its brevity and conceptual clarity. While no formal cultural adaptation was performed, item language was reviewed for relevance and sensitivity to local professional norms.

#### Experiences questionnaire

Experiences Questionnaire (Fresco & Mennin, 2019) was used to measure to the ability to psychologically detach from work after working hours. The scale has 20 items rated on a 5-point Likert scale from 1 (never) to 5 (all the time). The detachment subscale, used in this study, aligns with theories of psychological boundary management and has been used in prior cross-cultural studies of occupational stress. No language or item modifications were made, as the items were deemed applicable without cultural adaptation in the professional English-speaking sample. The detachment subscale exhibited strong internal consistency in our sample with a Cronbach’s alpha 0f .83.

#### Secondary Traumatic Stress Scale

Secondary traumatic stress symptoms were measured using the Secondary Traumatic Stress Scale (STSS) was developed (Bride et al., 2004). This 17-item scale captures symptoms of intrusion, avoidance, and arousal mirroring DSM-IV PTSD criteria experienced due to indirect exposure to trauma through work with traumatized clients. Participants rated each item on a scale from 1 (never) to 5 (very often), referring to experiences over the past 7 days.

The STSS was used in this study as the primary instrument for measuring STS, as it provides a more comprehensive and clinically grounded assessment. Scoring follows standard cutoffs: Score 27 or less indicate little or no STS, score 28 to 37 = mild, score 38 to 43 = moderate STS, 44 to 48 = high STS, and score 49+ = severe STS (Bride et al., 2004).

The STSS has been validated in diverse contexts and was chosen for its clinical specificity. Although no formal cultural adaptation was conducted, the English version was used among professionals proficient in English in Pakistan. The STSS demonstrated excellent internal consistency in this study (Cronbach’s alpha = .95), with high reliability on its subscales: intrusion (α = .86), avoidance (α = .88), and arousal (α = .88; Ting et al., 2005).

### Statistical analysis

The statistical analyses were conducted using SPSS version 29. Descriptive statistics were used to summarize the demographic characteristics of the sample, such as gender, age, marital status, education, occupation, and work experience with refugees. To assess the prevalence level of STS among participants, scores were calculated using the STS scale. This scale categorizes participants into five groups based on their scores: those experiencing little to no STS, mild STS, moderate STS, high STS, and severe STS.

Regression analyses were performed to examine the relationship between burnout, age, detachment, working hours, and STS. The goal was to determine whether burnout and age predict STS, and how detachment and working hours moderate the relationship between burnout and STS. Before conducting regression analyses, key assumptions of normality, linearity, homoscedasticity, and independence of errors were tested using residual plots and statistical tests (e.g. Kolmogorov–Smirnov test and Durbin–Watson statistic). Multicollinearity was assessed using Variance Inflation Factor (VIF) and tolerance values; all VIF values were below the recommended threshold of 5.

Furthermore, Moderation analyses were carried out using interaction terms between burnout and the moderators (detachment and working hours). Prior to creating interaction terms, all continuous predictor and moderator variables were mean-centered to reduce multicollinearity and improve interpretability of the interaction effects. Interaction terms were computed by multiplying the centered burnout variable with each centered moderator variable.

Bootstrapping with 5,000 samples was applied to test the significance of moderation effects, with confidence intervals reported at the 95% level. Mediation analysis followed the approach of Baron and Kenny, and the indirect effects were tested using bootstrapped confidence intervals to assess the mediating role of burnout between age and STS.

Additionally, mediation analyses were conducted to test whether burnout mediates the relationship between age and STS. Mediation and moderation analyses were conducted using SPSS PROCESS macro.

## Results

The primary aim of this study was to investigate the relationship between burnout, STS, and factors such as age, detachment, and working hours among mental health professionals working with war refugees in Pakistan. To explore these relationships, we conducted several analyses, including regression, moderation, and mediation analyses, to understand both direct and indirect effects of these variables.

### Prevalence of STS among participants

The first step was to examine the prevalence of STS among participants, as shown in Table 2. The findings indicate that 63 out of 103 participants (61.2%) reported experiencing STS, while 40 participants (38.8%) did not.

**Table 2.** Prevalence of STS among participants (*n* = 103).

STS	Frequency	Percentage (%)
Yes	63	61.2
No	40	38.8
Total	103	100

These results indicate that a substantial proportion (61.2%) of mental health professionals working with refugees are experiencing symptoms of STS, highlighting the occupational risk associated with providing care to this population.

Next, the severity of STS symptoms was examined, revealing a distribution across different levels, as shown in Table 3. While 37.9% of participants reported no symptoms of STS, 28.2% experienced mild symptoms, and 13.6% reported moderate symptoms. Notably, 12.6% experienced high STS, and 7.8% had severe STS.

**Table 3.** Level of STS among participants (*n* = 103).

STS level	Frequency	Percentage (%)
No STS	39	37.9
Mild	29	28.2
Moderate	14	13.6
High	13	12.6
Severe	8	7.8
No STS	39	37.9

The distribution of STS severity shows that while a portion of the sample reported no symptoms, a significant number of participants (34%) reported moderate to severe STS symptoms, reflecting a notable psychological burden among professionals in this context.

### Relationship between burnout, detachment, and STS

To examine the relationship between burnout, detachment, and STS, a regression model was constructed using burnout and detachment as predictor variables and STS as the outcome variable. The analysis revealed that the model explained 49% of the variance in STS (*R*^2^ = .49) and was statistically significant, indicating a good fit to the data (*F*(2, 103) = 52.39, *p* < .001).

Burnout was found to be a significant positive predictor of STS (β = .71, *p* < .001), indicating that higher levels of burnout were associated with increased STS among mental health professionals serving war refugees. In contrast, detachment did not significantly predict STS (β = .01, *p* = .550), suggesting that emotional detachment alone does not mitigate STS directly. These findings are summarized in Table 4.

**Table 4.** Regression analysis results for predicting STS from burnout and detachment.

Predictor	β	*SE*	β	*t*	*p*
Constant	.71	0.055	–	12.91	<.001
Burnout	.71	0.099	.71	7.18	<.001
Detachment	.01	0.020	.01	0.60	.550

*Note*. The model was statistically significant, $R^{2}=0.49,\;\;\;\;F\left(2,103\right)=52.39,\;\;p<0.001$ . Bootstrapping with 5,000 resamples was applied to improve confidence intervals. Standardized coefficients (β) are reported. *p* < .05 is considered significant.

Table 4 reports that Higher burnout significantly predicted greater STS, while detachment showed no significant direct effect on STS in this model.

### Moderation of burnout-STS relationship by detachment and work hours

The next aim of the study was to examine whether detachment and work hours moderated the relationship between burnout and STS. Two moderation analyses were conducted to address this question.

The first moderation analysis tested the hypothesis that detachment would buffer the effect of burnout on STS. The model was constructed using burnout as the independent variable, STS as the dependent variable, and detachment as the moderator. The interaction term between burnout and detachment was statistically significant and the moderation effect had a small-to-moderate effect size meaning the impact of burnout on STS was weaker among those reporting higher detachment (β = −.16, *p* = .034), indicating that detachment significantly moderated the relationship between burnout and STS. The interaction suggests that while burnout increased STS, the impact was weaker among participants who reported higher levels of detachment (see Table 5).

**Table 5.** Moderation analysis of detachment as a moderator between burnout and *STS*.

Predictor variable	Unstandardized coefficient (*B*)	*p-*Value	95% Confidence interval (lower)	95% Confidence interval (upper)
Burnout (independent variable)	1.78	<.001	1.40	2.16
Detachment (moderator)	−0.09	.657	−0.52	0.31
Burnout × detachment (interaction term)	−0.06	.034	0.01	0.11

*Note.* A moderation analysis was conducted to examine whether Detachment moderated the relationship between burnout and STS. Independent variable: Burnout. Moderator: Detachment. Interaction term: Burnout × Detachment. *B* = unstandardized regression coefficient; CI = confidence interval.

These results indicate that Detachment buffered the relationship between burnout and STS, meaning that individuals with higher detachment experienced a weaker impact of burnout on their STS symptoms.

In the second moderation analysis, we evaluated whether the number of work hours spent with refugees moderated the relationship between burnout and STS. The interaction term was also statistically significant and the standardized interaction effect was moderate showing a greater risk of STS with increased exposure to burnout in high-contact roles (β = .18, *p* = .005), indicating that the relationship between burnout and STS was stronger for individuals working longer hours with refugees (Table 6). This suggests that professionals who spend more time directly engaged with refugee populations are more vulnerable to the effects of burnout on STS.

**Table 6.** Moderation analysis of work hours as a moderator between burnout and STS.

Predictor variable	Unstandardized coefficient (*B*)	*p*-Value	95% confidence interval (lower)	95% confidence interval (upper)
Burnout (independent variable)	2.705	<.001	1.951	3.459
Work hours (moderator)	1.002	.007	0.290	1.714
Burnout × work hours (interaction term)	0.039	.005	−0.066	−0.012

*Note.* A moderation analysis was conducted to examine whether Work Hours moderated the relationship between burnout and STS. Independent variable: Burnout. Moderator: Work Hours. Interaction term: Burnout × Work Hours. *B* = unstandardized regression coefficient; CI = confidence interval.

It is evident from results above that the relationship between burnout and STS was stronger for professionals working more hours with refugees, indicating that increased exposure amplifies the negative impact of burnout on STS.

Both moderation analyses were conducted using 5,000 bootstrapped samples to enhance the accuracy of the interaction effects, particularly given the relatively small sample size.

### Age as a predictor of burnout

To further explore potential predictors of burnout, we investigated whether age had a significant impact. A separate regression model was constructed with burnout as the outcome variable and age as the predictor. The results, presented in Table 7, show that age was a significant negative predictor of burnout (*B* = −.140, *p* = .020), indicating that as age increases, burnout tends to decrease. This suggests that older professionals may have more experience managing work-related stress, resulting in lower burnout levels. The model explained approximately 4.9% of the variance in burnout (*R*^2^ = .049), and the standardized coefficient (β = −.221) reflects a moderate effect size.

**Table 7.** Regression analysis predicting burnout from age.

Predictor	*B*	*SE*	β	*t*	*p*
Constant	30.945	2.122	–	14.584	<.001
Age	−0.140	0.059	−.221	−2.367	.020

*Note*. This model evaluates age as a predictor of burnout. Standardized regression coefficients (β) are reported. *p* < .05 is considered significant.

Table 7 shows that Older professionals reported lower levels of burnout, suggesting that greater age may be associated with improved coping or resilience in managing work-related stress.

### Mediation of age and STS by burnout

Finally, a mediation analysis was conducted to explore whether burnout mediated the relationship between age and STS. The results indicate that age had a significant indirect effect on STS through burnout (Estimate = −0.2401, *p* = .020), suggesting that as individuals age, their likelihood of experiencing burnout decreases, which in turn reduces their STS. However, the direct effect of age on STS was not significant (*p* = .501), indicating that age alone does not directly influence STS without considering burnout. The total effect was also not significant (*p* = .240), confirming that burnout acts as a mediator in the relationship (Table 8). Effect sizes for mediation paths were also calculated. Age had a moderate negative effect on burnout (β = −.40), burnout had a strong effect on STS (β = −.50), and the indirect effect was moderate (β = −.24)

**Table 8.** Mediation model of burnout, age, and secondary traumatic stress symptoms (STSS).

Variables	Effect	*B*	*SE*	*p*	95% CI		%
					LL	UL	
Age (X)	Total	−0.30	0.14	.240	−0.57	0.03	100
	Direct	−0.10	0.10	.501	−0.29	0.09	5.67
	Indirect	0.20	0.09	.020	0.02	0.38	94.33
Burnout (M)	Effect on M	−0.40	0.10	<.001	−0.60	−0.20	100
STS (Y)	Effect on Y	−0.50	0.12	<.001	−0.74	−0.26	100

*Note*. X = independent variable; M = mediator; Y = dependent variable.

Table 8 explains that Burnout was found to mediate the relationship between age and STS, indicating that older professionals tend to experience less burnout, which in turn reduces their risk of developing STS.

### Moderation model

**Figure 1.**
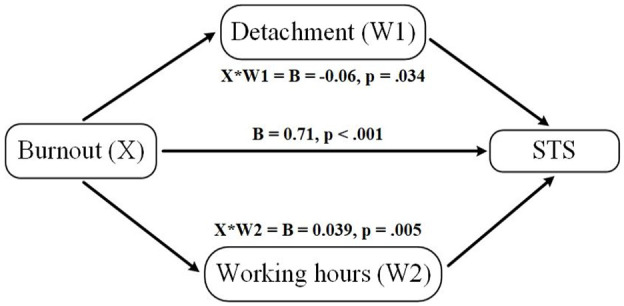
Moderation model of burnout, detachment, working hours, and STS. *Note.* The model illustrates the moderating effects of detachment and weekly work hours on the relationship between burnout and STS. Burnout is the independent variable (X), STS is the dependent variable (Y). Detachment (W1) and work hours (W2) are moderators of the Burnout → STS relationship. Higher detachment buffers (weakens) the positive association between burnout and STS, while longer work hours amplify this association. Arrows indicate hypothesized directional relationships.

The model presented in Figure 1 illustrates the relationships among burnout, detachment, work hours, and STS. This model hypothesizes that detachment and work hours moderate the effect of burnout on STS. The results from the study confirmed this hypothesized model, providing support for the proposed relationships.

### Mediation model

**Figure 2.**
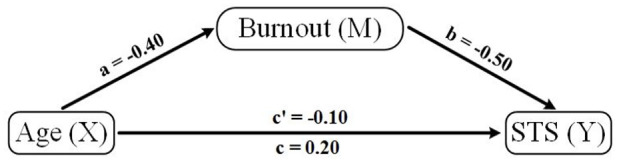
Mediation model of age, burnout, and STS. *Note*. The model depicts burnout as a mediator of the relationship between age and STS. Age is the independent variable (X), burnout is the mediator (M), and STS is the dependent variable (Y). As age increases, burnout decreases, which in turn reduces STS levels. The path labeled c’ represents the direct effect of age on STS (non-significant), while the path through burnout represents the significant indirect effect (c path), suggesting that burnout explains the link between age and STS.

The model given in Figure 2 establishes the relation between age, burnout, and STS. It was hypothesized that burnout will act as a mediator between age and STS. Mediation analysis showed that burnout mediated the relationship between age and STS, with age reducing burnout and, in turn, STS (*p* = .020). These results validate the proposed model, emphasizing the need to address burnout to mitigate STS.

## Discussion

The findings of this study provide valuable insights into the relationships between burnout, STS, detachment, work hours, and age among mental health professionals working with war refugees in Pakistan. This investigation has revealed several critical findings that offer insights into how these factors interact and influence the well-being of individuals providing care to war refugees. Understanding these relationships is crucial for developing interventions aimed at mitigating STS and improving the well-being of professionals who provide care in such challenging environments.

Our study confirmed that burnout is a significant predictor of STS among mental health professionals working with refugees. This finding aligns with existing research, which consistently shows that prolonged exposure to trauma-exposed populations increases professionals’ susceptibility to burnout (Bride, 2007). Burnout, characterized by emotional exhaustion and depersonalization, impairs the ability to cope with the emotional strain of working with trauma survivors, such as refugees. Previous studies have similarly linked burnout to emotional contagion in caregiving professions, where professionals absorb their clients’ emotional burdens, leading to psychological distress (Cummings et al., 2021; Hamama et al., 2019). Research has also demonstrated that extended exposure to trauma heightens the risk of emotional exhaustion and psychological distress, which intensifies burnout’s effects (Bride, 2007; Collins & Long, 2003; Melese et al., 2024). It further identifies burnout as a key predictor of STS, reinforcing the critical need to address burnout through targeted interventions. Given the high levels of burnout in refugee care settings, implementing strategies to reduce burnout is essential for mitigating the risk of STS development.

Furthermore, this study highlights that the number of hours spent working with refugees acts as a crucial factor in moderating the relationship between burnout and STS. Our findings indicate that professionals who engage with refugees for longer hours daily are more likely to experience heightened levels of STS as their burnout intensifies. This result aligns with previous research, which emphasizes that the intensity of contact (work hours) significantly contribute to the development of burnout and STS (Hamama et al., 2019). The cumulative nature of trauma exposure suggests that more extended work hours lead to more frequent encounters with distressing experiences, exacerbating burnout and its associated consequences. Our results show a stronger positive relationship between burnout and STS among professionals who spend more hours working with refugees, further underscoring the emotional burden that comes with prolonged and intense exposure (Bride, 2007). This finding highlights the pressing need for better workload management, protective strategies, and work-hour regulation in such high-stress environments. Implementing measures such as reducing work hours, introducing regular breaks, or rotating responsibilities could be critical in protecting mental health professionals from the adverse effects of prolonged trauma exposure.

Interestingly, detachment did not directly predict STS in our sample, suggesting that in isolation, the capacity to emotionally distance oneself from work-related stressors may not be sufficient to buffer the effects of trauma. However, this finding stands in contrast to previous studies that have identified detachment as a protective factor against STS. One possible explanation for this discrepancy may lie in cultural differences in how detachment is perceived or practiced among Pakistani professionals. In collectivist cultures, emotional distancing from clients may be viewed less favorably or may not be widely used as a coping strategy. Job role expectations in local contexts may also discourage emotional disengagement, especially in caregiving roles, where empathy and compassion are emphasized. Additionally, the measurement tools used to assess detachment may lack cultural sensitivity or specificity, potentially limiting their accuracy in capturing the construct. These factors could contribute to the non-significant direct relationship observed in this study. Moreover, the pattern of results may reflect the complex nature of detachment as a coping strategy. Detachment is often conceptualized as an emotion-focused coping strategy aimed at protecting emotional resources (Folkman & Lazarus, 1988). In this role, detachment may not directly prevent the experience of STS, especially in response to acute traumatic exposure, but may buffer against the cumulative emotional exhaustion that underlies the burnout → STS pathway (Rupert et al., 2015). Prior literature suggests that coping strategies can vary in their direct versus indirect effects on well-being outcomes, with some strategies more effective at managing chronic stressors like burnout than at reducing acute trauma-related symptoms (Ben-Zur, 2016). The current findings align with this perspective: while detachment did not directly reduce STS, it moderated the burnout → STS link, potentially by preserving emotional energy and reducing susceptibility to stress amplification under conditions of high burnout. This interpretation highlights the need to consider detachment within a broader taxonomy of coping strategies, recognizing that its protective effects may operate through interaction with other stress processes rather than through a direct effect on trauma symptoms themselves.

However, detachment did play a significant role as a moderator between burnout and STS. Specifically, our results indicate that higher levels of detachment mitigate the impact of burnout on STS. This finding aligns with Lazarus and Folkman’s Transactional Model of Stress and Coping, which posits that coping strategies vary in effectiveness depending on the type of stressor (Provencher, 2007). Detachment, as an emotion-focused coping strategy, may be more effective in managing chronic stress like burnout, which develops over time and erodes emotional resources (Kotera et al., 2021). Burnout, characterized by prolonged emotional exhaustion and cynicism, can leave individuals more vulnerable to the negative effects of secondary traumatic stress. By emotionally distancing themselves from work-related stressors, individuals are better able to preserve their emotional resources, preventing burnout from escalating into more severe stress reactions, such as STS (Rupert et al., 2015). This could explain why higher levels of detachment helped to reduce the impact of burnout on STS in our sample.

In contrast, acute stress like trauma exposure requires different coping strategies, such as problem-solving or seeking support (Ben-Zur, 2019). The transactional model emphasizes that coping strategies must match the nature of the stressor to be effective. Detachment may not be as effective in directly reducing STS because secondary trauma is often an immediate and intense emotional experience, where emotional distancing might not provide adequate protection. Instead, trauma may require active engagement and processing, rather than disengagement. This distinction could help explain why detachment did not directly predict STS in our study but was effective in buffering the relationship between burnout and STS.

In terms of age, our study addresses an important research gap regarding the relationship between age and STS. While existing literature indicates that older professionals tend to be less susceptible to STS, the specific mechanisms underlying this relationship remain unclear (Craig & Sprang, 2010; De et al., 2009). Our findings provide valuable insights by suggesting that burnout may serve as a potential explanatory link between age and STS. Specifically, we observed a significant statistical indirect effect of age on STS through burnout. While this pattern is consistent with a mediation model in which lower burnout among older professionals may help explain lower STS, the cross-sectional nature of the data prevents us from inferring causal or temporal relationships. Future longitudinal research is needed to confirm the directionality of these effects. Several past findings also support this (O’Connor et al., 2018). This pattern may reflect a few mechanisms. Older professionals may have developed stronger coping strategies over time, such as cognitive reframing, emotional regulation, and boundary-setting, making them less vulnerable to burnout. Emotional desensitization, while not always adaptive, may also occur with prolonged professional exposure to trauma, thereby reducing reactivity to stressful situations.

This suggests that older mental health professionals may possess more resilience or adaptive coping mechanisms to deal with the emotional demands of working with refugees, likely due to accumulated experience and better emotional regulation (Mendes & Miguel, 2024). This finding is consistent with research suggesting that older mental health professionals often have more well-developed coping strategies and emotional regulation skills compared to their younger counterparts (O’Connor et al., 2018). While age did not directly affect STS, its influence through burnout highlights the importance of burnout prevention strategies across all age groups, particularly for younger professionals who may be more vulnerable to burnout.

The context of this study mental health professionals serving war refugees in Pakistan presents unique challenges, as these professionals navigate the complexities of trauma, displacement, and uncertainty faced by their clients. This emotional burden underscores the urgent need for robust organizational support systems and tailored interventions aimed at enhancing the well-being of caregivers. Developing targeted strategies to mitigate burnout, promote detachment, and effectively manage workloads could significantly reduce STS within this population. Organizations can promote psychological detachment by encouraging staff to maintain boundaries between work and personal life, offering training in boundary-setting and self-care, and providing regular supervision and debriefing opportunities. Structuring work schedules to allow adequate recovery time can further support professionals’ ability to detach and manage stress. Future research should consider cross-cultural validation of coping constructs like detachment and examine how cultural norms and workplace expectations influence the adoption of such strategies. Future research should also explore the implementation and effectiveness of such interventions, as well as examine the broader implications of organizational culture and support on mental health professionals in similar high-stress environments.

## Limitations

This study has several limitations that should be acknowledged. Firstly, the sample size was relatively small, which may limit the generalizability of the findings to a broader population of mental health professionals working with war refugees. Moreover, moderation analyses particularly interaction effects are known to require larger sample sizes for sufficient statistical power and robust detection. Thus, the findings related to moderation effects should be interpreted with caution. Additionally, the use of an online survey presented challenges in reaching participants, resulting in a low response rate. Furthermore, the cross-sectional nature of the study does not allow for causal inferences to be drawn regarding the relationships between burnout, detachment, and STS. Future research would benefit from larger, more representative samples, and longitudinal designs to better understand these dynamics over time.

Another important limitation is the limited generalizability of these findings beyond the specific sociocultural and healthcare context of Pakistan. Factors such as cultural norms, institutional support systems, and stigma around mental health may differ significantly in other regions, which could influence the applicability of the results across countries.

Lastly, while the survey aimed to assess key variables, self-report measures are inherently subject to biases, such as social desirability and recall bias, which may influence participants’ responses and affect the accuracy of the findings. Future studies should consider incorporating multi-method data collection approaches, including observational or peer-report measures, to reduce this risk. Addressing these limitations in future studies will be crucial for advancing the understanding of STS among mental health professionals in high-stress environments.

## Conclusion

The findings of this study emphasize the urgent need for organizational and policy-level interventions that directly target burnout, particularly among younger professionals and those with high trauma exposure due to extended work hours. Support strategies should include structured recovery protocols, caseload redistribution, and specialized training in emotion-focused coping techniques such as mindful detachment. Promoting a healthy work-life balance and offering routine supervision could also help reduce the psychological burden. Future research should explore these intervention strategies in longitudinal and cross-cultural settings to assess their effectiveness over time and across different professional populations. Additionally, future studies should employ longitudinal designs to more rigorously test potential causal pathways between age, burnout, and STS, as cross-sectional data cannot establish causality.
